# Essential roles of oncostatin M receptor β signaling in renal crystal formation in mice

**DOI:** 10.1038/s41598-020-74198-3

**Published:** 2020-10-13

**Authors:** Shimpei Yamashita, Tadasuke Komori, Yasuo Kohjimoto, Atsushi Miyajima, Isao Hara, Yoshihiro Morikawa

**Affiliations:** 1 Department of Urology, Wakayama Medical University, Wakayama, Japan; 2 Department of Anatomy and Neurobiology, Wakayama Medical University, 811-1 Kimiidera, Wakayama, 641-8509 Japan; 3 Laboratory of Cell Growth and Differentiation, Institute for Quantitative Biosciences, The University of Tokyo, Tokyo, Japan

**Keywords:** Kidney diseases, Renal calculi

## Abstract

Oncostatin M (OSM), a member of the IL-6 family of cytokines, has important roles in renal diseases. The relationship between OSM and kidney stone disease, however, remains unclear. To investigate the roles of OSM in the development of kidney stone disease, we generated a mouse model of renal crystal formation using OSM receptor β (OSMRβ)-deficient mice (OSMRβ^−/−^ mice). There were fewer renal crystal deposits in OSMRβ^−/−^ mice than in wild-type (WT) mice. Crystal-binding molecules (osteopontin, annexin A1, and annexin A2), inflammatory cytokines (TNF-α and IL-1β), and fibrosis markers (TGF-β, collagen 1a2, and α-smooth muscle actin) were also decreased in the kidneys of OSMRβ^−/−^ mice compared with those in WT mice. Immunofluorescence staining showed that OSMRβ was expressed in renal tubular epithelial cells (RTECs) and renal fibroblasts in the model of renal crystal formation. In the cultured RTECs and renal fibroblasts, OSM directly induced the expression of crystal-binding molecules and fibrosis markers. Expressions of inflammatory cytokines were increased by stimulation with OSM in cultured renal fibroblasts. OSM may promote the formation of renal crystal deposits by directly acting on RTECs and renal fibroblasts to produce crystal-binding molecules and inflammatory cytokines.

## Introduction

Kidney stone disease, also known as urolithiasis, is a common urologic disorder and is associated with the development of chronic kidney disease (CKD) and end-stage renal disease^1–3^. Over the past 30 years, management of kidney stone disease based on the stone removal has evolved to include minimally invasive approaches, such as shock wave lithotripsy and ureteroscopic fragmentation and retrieval^1^. Meanwhile, approximately 50% of patients with kidney stones have recurrence of stones within 5–10 years after a first renal stone^1,4–6^. Once recurrent, the interval between stone formations is shortened^4,7^. Prevalence of kidney stone disease has, therefore, steadily increased in most countries^1,8,9^, including the United States^10^ and Japan^11^. From this point of view, the prevention of stone formation in addition to stone disintegration is important in the treatment of kidney stone disease.

Approximately 80% of kidney stones are composed of calcium oxalate (CaOx)^1,8,11^. In the urinary tract, stone formation begins by the supersaturation of stone constituents in the urine, leading to the crystallization of CaOx. Crystals grow, aggregate, and adhere to renal tubular cells, and are retained in the tubules^1,8^. However, the supersaturation of urine with respect to CaOx^12^ and the formation of small particles of CaOx crystal^13^ often occur even in the healthy people. As growth of CaOx crystals is slow, it is unlikely that single crystals grow large enough to be lodged in the terminal collecting duct of the kidney within typical urine transit time^14,15^. Consequently, critical processes in stone formation are represented by crystal aggregation, attachment of crystals or aggregates to renal epithelial cells, and crystal retention.

Some cytokines were recently reported to involve attachment of crystals or aggregates to renal epithelial cells, and crystal retention. Mulay et al.^16^ reported that in tubular epithelial cells, tumor necrosis factor receptor (TNFR) signaling induces the expression of crystal adhesion molecules, CD44 and annexin (ANX) II. The treatment of hyperoxaluric mice with TNFR inhibitor partially prevents crystals from aggregating and adhering^16^. Furthermore, Taguchi et al.^17^ demonstrated that colony stimulating factor-1 (CSF-1)-deficient mice had marked increase in renal crystal deposition. The reintroduction of M2 (anti-inflammatory) macrophages by CSF-1 treatment reduce renal crystal deposition and CaOx crystal formation^17^. In addition, they reported that renal crystal development is facilitated by M1 (inflammatory) macrophages^18^. These findings suggest that cytokines involved with inflammation and macrophage phenotypic switch play some important roles in crystal aggregation, adhesion, and retention, which are the critical steps of renal stone formation.

Oncostatin M (OSM), a member of the interleukin-6 (IL-6) family of cytokines, has been reported to play an important role in a variety of functions, such as hematopoiesis and heart remodeling^19^. In addition to these functions, OSM is also known to be involved in a variety of inflammatory diseases^20^. In several inflammatory diseases, such as pulmonary inflammation and inflammatory bowel diseases, OSM promotes inflammation^21,22^. In contrast, OSM suppresses adipose tissue inflammation by changing the phenotype of adipose tissue macrophages from M1 type (pro-inflammatory) to M2 type (anti-inflammatory)^23,24^. The roles of OSM in the development of kidney stone disease, however, remain unclear.

OSM receptor consists of gp130, a common receptor subunit to the IL-6 family of cytokines, and the OSM receptor β subunit (OSMRβ)^19,20^. The lack of OSMRβ has been reported to lead to adipose tissue inflammation and insulin resistance by switching the phenotype of macrophages from M2 type to M1 type^23^. To evaluate the roles of OSM in kidney stone disease, we examined the expressions of OSM and OSMRβ in an experimental model of kidney stone disease. We also investigated crystal formation, inflammatory status, and the expression of crystal-binding molecules in the kidney of OSMRβ-deficient (OSMRβ^−/−^) mice.

## Results

### Expressions of OSM and OSMRβ in the kidneys of a mouse model of renal crystal formation

**Figure 1:**
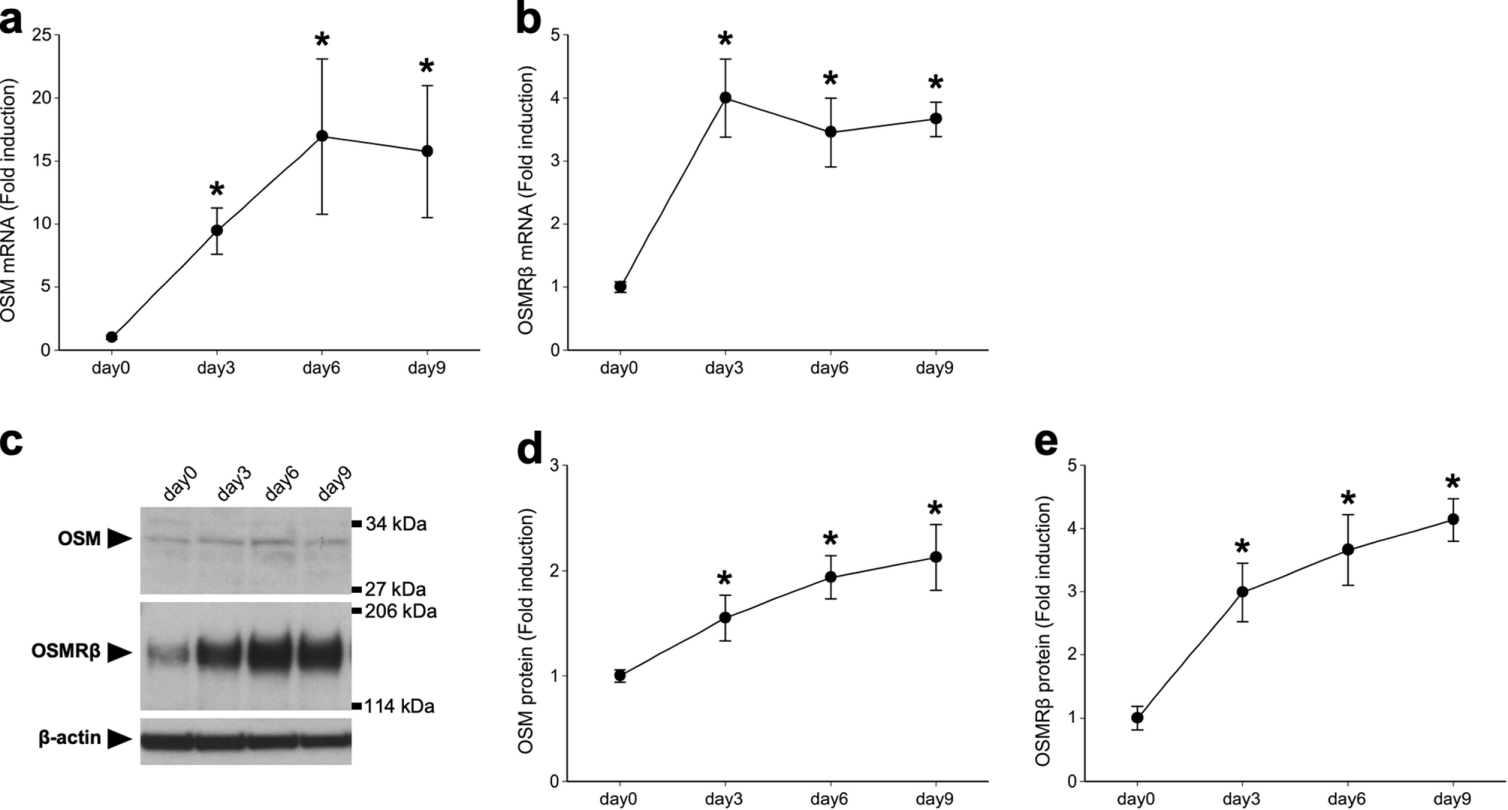
Expressions of OSM and OSMRβ in the kidney of a mouse model of renal crystal formation. C57BL/6 J mice were given intraperitoneal injection of GOx (80 mg/kg) once daily for three, six, or nine days. (**a**,**b**) Gene expression of OSM (**a**) and OSMRβ (**b**) in the kidney on days 0 (without GOx injection), 3, 6, or 9. (**c**) Protein expression of OSM and OSMRβ in the kidney on days 0, 3, 6, or 9. The apparent molecular weights are indicated on the right. (**d**,**e**) Quantitative analysis of the protein expressions of OSM and OSMRβ. The band intensities of OSM and OSMRβ were normalized to β-actin and represented as the fold induction relative to the intensities on day 0. The full-length blots are presented in Supplementary Fig. S4. Data are expressed as mean ± SEM; n = 4–7 per group. *P < 0.05 compared with day 0.

To speculate the roles of OSM in the pathogenesis of kidney stone disease, we first elucidated the expressions of OSM and OSMRβ in the kidney of glyoxylate (GOx)-injected wild-type (WT) mice, a mouse model of renal crystal formation. Quantitative real-time PCR analysis revealed that the mRNA expression of OSM began to increase on day 3 and peaked on day 6 (Fig. 1a). The expression of OSMRβ mRNA was increased and peaked on day 3 (Fig. 1b). The mRNA expressions of both OSM and OSMRβ then gradually decreased until day 9, but still remained higher than those on day 0 (Fig. 1a,b). Consistent with the expression patterns of these genes, Western blot analysis revealed that the protein expressions of OSM and OSMRβ were increased on days 3, 6, and 9 compared with those on day 0 (Fig. 1c–e).

### Formation of GOx-induced renal crystal deposits in OSMRβ^−/−^ mice

**Figure 2:**
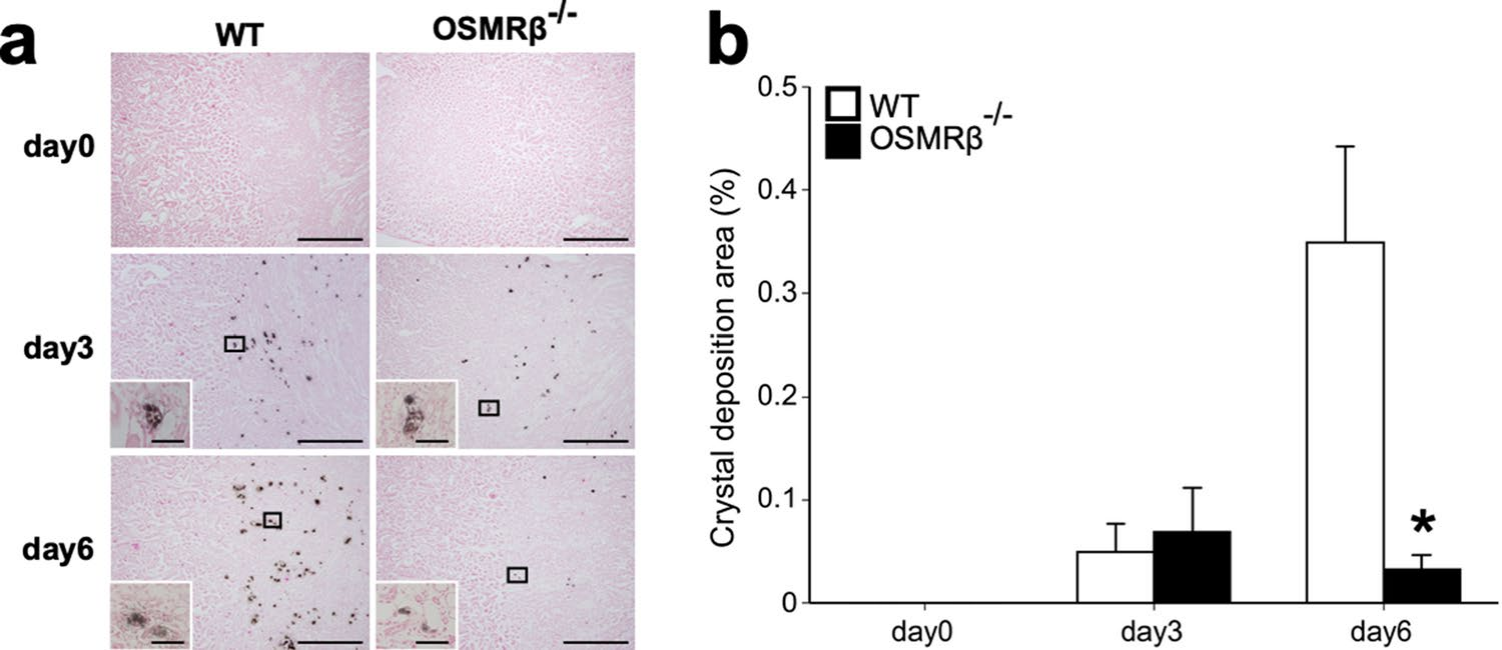
Renal crystal deposits in WT and OSMRβ^−/−^ mice. WT and OSMRβ^−/−^ mice were given intraperitoneal injection of GOx (80 mg/kg) once daily for three or six days. (**a**) Pizzolato staining in the kidney of WT and OSMRβ^−/−^ mice on days 0 (without GOx injection), 3, or 6. The boxed regions are shown at a higher magnification in the insets. Scale bars = 100 μm; 50 μm (insets). (**b**) The ratio of areas with renal crystal deposits. The ratio was quantified by calculating the percentage of the area containing crystal deposits to the total kidney area. Data are expressed as mean ± SEM; n = 6 per group. *P < 0.05 compared with WT mice.

To investigate the roles of OSM signaling in renal crystal formation, WT and OSMRβ^−/−^ mice were given intraperitoneal injection of GOx. On day 0, no renal crystal deposits were observed in either WT or OSMRβ^−/−^ mice (Fig. 2). On day 3, some crystal deposits were observed in the intratubular space of the kidney in both WT and OSMRβ^−/−^ mice (Fig. 2a). There were no differences in the amount of renal crystal deposits between WT and OSMRβ^−/−^ mice on day 3 (Fig. 2b). On day 6, renal crystal deposits were markedly increased in WT mice (Fig. 2). However, renal crystal deposits in OSMRβ^−/−^ mice were significantly decreased compared with those in WT mice on day 6 (Fig. 2).

### Expressions of crystal-binding molecules in the kidneys of OSMRβ^−/−^ mice

**Figure 3:**
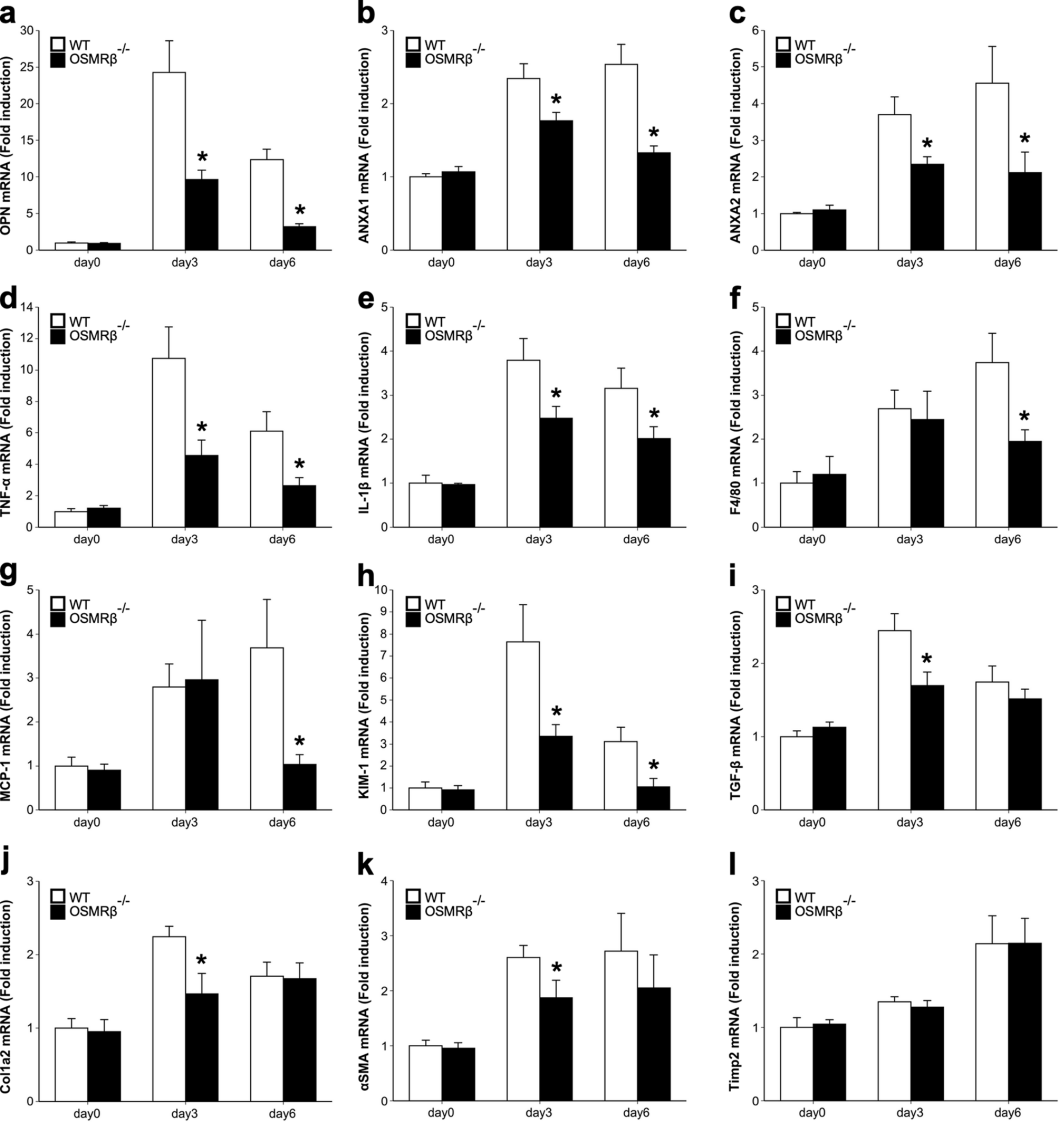
Gene expressions in the kidney of WT and OSMRβ^−/−^ mice after GOx injection. WT and OSMRβ^−/−^ mice were given intraperitoneal injection of GOx (80 mg/kg) once daily for three or six days. (**a**–**c**) Gene expressions of crystal-binding molecules, OPN (**a**), ANXA1 (**b**), and ANXA2 (**c**), in the kidney of WT and OSMRβ^−/−^ mice on days 0 (without GOx injection), 3, or 6. (**d**–**g**) Expressions of genes related to inflammation, TNF-α (**d**), IL-1β (**e**), F4/80 (**f**), and MCP-1 (**g**), in the kidney of WT and OSMRβ^−/−^ mice at days 0, 3, or 6. (**h**–**l**) Expressions of genes related to kidney injury, KIM-1 (**h**), and fibrosis, TGF-β (**i**), Col1a2 (**j**), αSMA (**k**), and Timp2 (**l**), in the kidneys of WT and OSMRβ^−/−^ mice on days 0, 3, or 6. Data are expressed as mean ± SEM; n = 5–7 per group. *P < 0.05 compared with WT mice.

**Figure 4:**
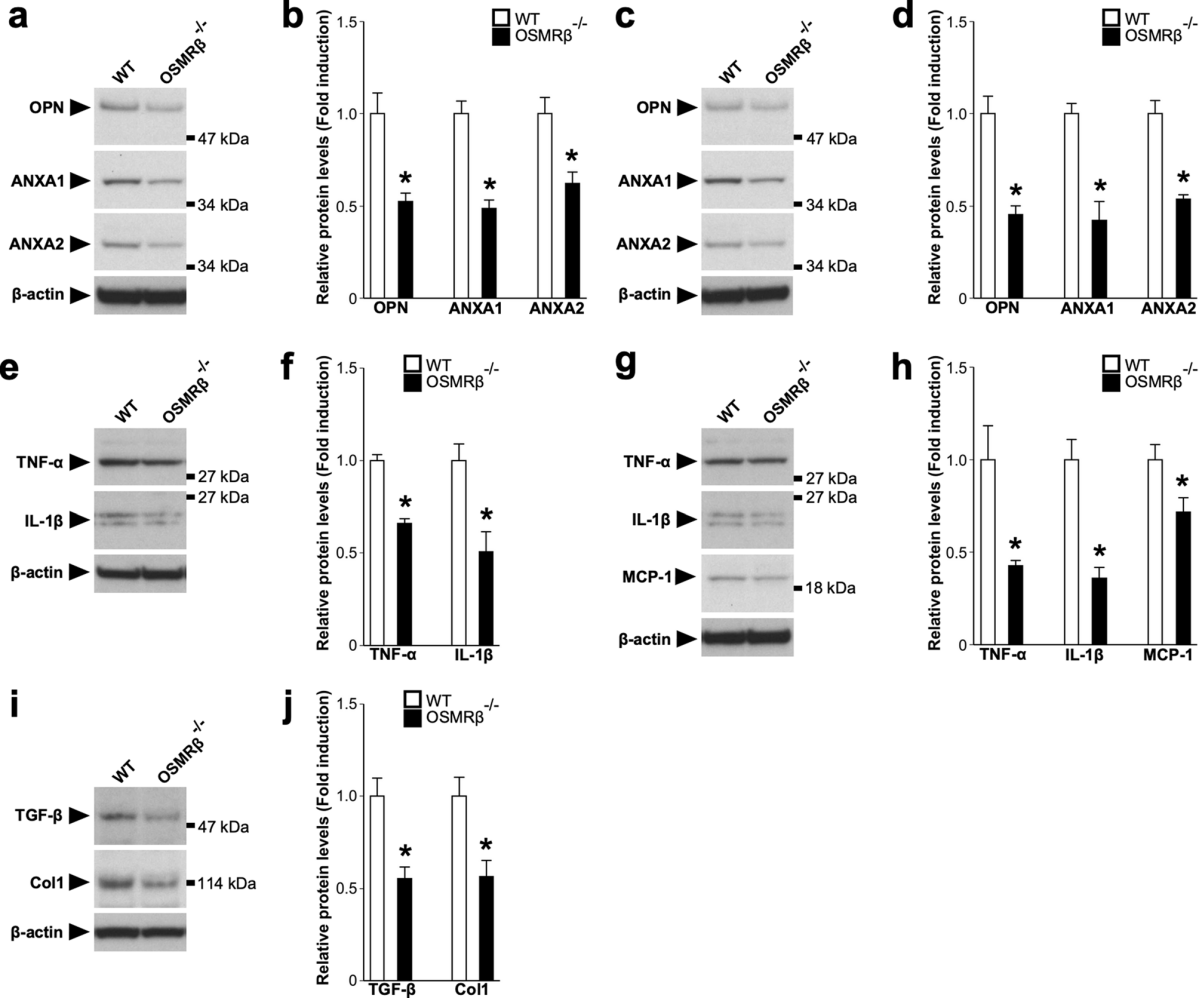
Protein expressions in the kidney of WT and OSMRβ^−/−^ mice after GOx injection. WT and OSMRβ^−/−^ mice were given intraperitoneal injection of GOx (80 mg/kg) once daily for three or six days. (**a**,**c**) Western blot analysis of crystal-binding molecules, OPN, ANXA1, and ANXA2, in the kidney of WT and OSMRβ^−/−^ mice on day 3 (**a**) and day 6 (**c**). The apparent molecular weights are indicated on the right. (**b,d**) Quantitative analysis of the protein expressions of OPN, ANXA1, and ANXA2 on day 3 (**b**) and day 6 (**d**). The band intensities of OPN, ANXA1, and ANXA2 were normalized to β-actin and are represented as the fold induction relative to the intensities of WT mice in the bar graph. (**e**,**g**) Western blot analysis of inflammation-related molecules, TNF-α (**e**,**g**), IL-1β (**e**,**g**), and MCP-1 (**g**), in the kidney of WT and OSMRβ^−/−^ mice on day 3 (**e**) day 6 (**g**). The apparent molecular weights are indicated on the right. (**f**,**h**) Quantitative analysis of the protein expressions of TNF-α (**f**,**h**), IL-1β (**f**,**h**), and MCP-1 (**h**) on day 3 (**f**) and day 6 (**h**). The band intensities of TNF-α, IL-1β, and MCP-1 were normalized to β-actin and are represented as the fold induction relative to the intensities of WT mice in the bar graph. (**i**) Western blot analysis of fibrosis-related molecules, TGF-β and Col1, in the kidney of WT and OSMRβ^−/−^ mice on day 3. The apparent molecular weights are indicated on the right. (**j**) Quantitative analysis of the protein expressions of TGF-β and Col1. The band intensities of TGF-β and Col1 were normalized to β-actin and are represented as the fold induction relative to the intensities of WT mice in the bar graph. The full-length blots are presented in Supplementary Fig. S5. Data are expressed as mean ± SEM; n = 4 per group. *P < 0.05 compared with WT mice.

To investigate the roles of OSM signaling in the expressions of crystal-binding molecules after GOx injection, we elucidated the expressions of osteopontin (OPN), ANXA1, and ANXA2 in the kidneys of WT and OSMRβ^−/−^ mice. On day 0, there were no differences in the mRNA expressions of these crystal-binding molecules between WT and OSMRβ^−/−^ mice (Fig. 3a–c). In WT mice, the mRNA expressions of OPN, ANXA1, and ANXA2 were increased on day 3 and day 6 compared with those on day 0 (Fig. 3a–c). The mRNA expressions of these crystal-binding molecules in OSMRβ^−/−^ mice were significantly suppressed, compared with those in WT mice on day 3 and day 6 (Fig. 3a–c). In addition, Western blot analysis confirmed the significant suppression of protein expressions of these crystal-binding molecules in OSMRβ^−/−^ mice on day 3 (Fig. 4a,b) and day 6 (Fig. 4c,d).

### GOx-induced inflammation in the kidney of OSMRβ^−/−^ mice

**Figure 5:**
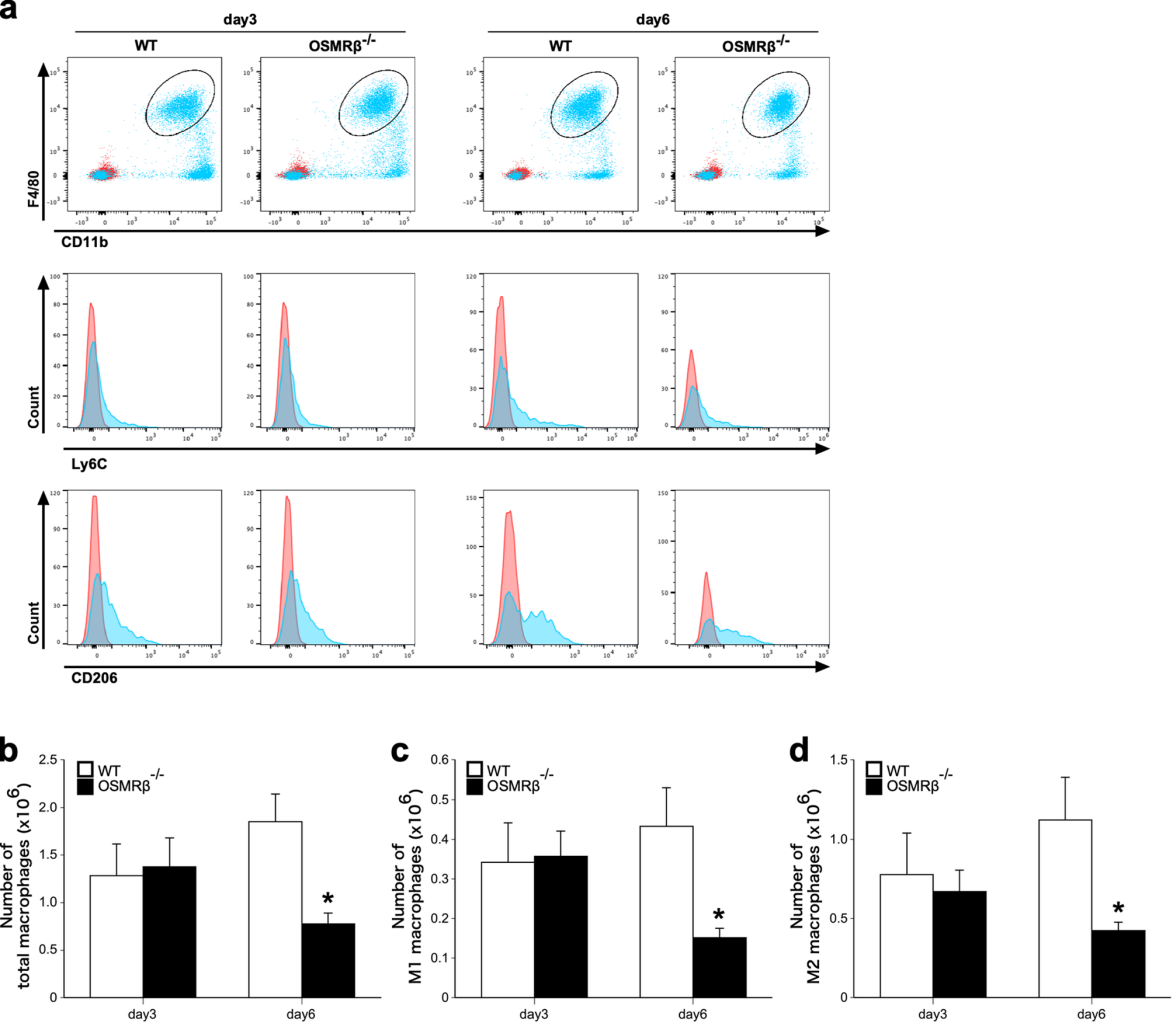
Flow cytometric analysis of renal macrophages in the kidney of WT and OSMRβ^−/−^ mice. WT and OSMRβ^−/−^ mice were given intraperitoneal injection of GOx (80 mg/kg) once daily for three or six days. (**a**) Dot plots of total macrophages (upper panels) and histogram plots of M1 (middle panels) and M2 (lower panels) macrophages in the kidney of WT and OSMRβ^−/−^ mice on day 3 and day 6. The isotype controls are shown as red dots (upper panels) and red areas (middle and lower panels). (**b**–**d**) The number of CD45^+^/F4/80^+^/CD11b^+^ cells (total macrophages; b), CD45^+^/F4/80^+^/CD11b^+^/Ly6C^+^ cells (M1 macrophages; c), and CD45^+^/F4/80^+^/CD11b^+^/CD206^+^ cells (M2 macrophages; d) in the kidney of WT and OSMRβ^−/−^ mice was evaluated by flow cytometric analysis. Data are expressed as mean ± SEM; n = 4–5 per group. *P < 0.05 compared with WT mice.

Next, we examined the inflammatory status in the kidney after GOx injection, because inflammation is an important factor in the development of kidney stone disease^18^. The injection of GOx induced the mRNA expressions of inflammatory cytokines (TNF-α and IL-1β), a marker of macrophages (F4/80), and a macrophage chemotactic factor (monocyte chemoattractant protein-1, MCP-1) in the kidneys of WT mice on day 3 and day 6 (Fig. 3d–g). On day 6, the mRNA expressions of TNF-α, IL-1β, F4/80, and MCP-1 in OSMRβ^−/−^ mice were significantly suppressed, compared with those in WT mice (Fig. 3d–g). In addition, on day 3, the mRNA expressions of TNF-α and IL-1β were also significantly decreased in OSMRβ^−/−^ mice compared with those in WT mice (Fig. 3d,e), but there was no difference in the expressions of F4/80 and MCP-1 between WT and OSMRβ^−/−^ mice (Fig. 3f,g). Furthermore, significant suppression of TNF-α, IL-1β and MCP-1 in OSMRβ^−/−^ mice was confirmed at the protein level (Fig. 4e–h). Flow cytometric analysis showed no significant differences in the numbers of total (CD45^+^/CD11b^+^/F4/80^+^ cells), M1 (CD45^+^/CD11b^+^/F4/80^+^/Ly6C^+^ cells), and M2 macrophages (CD45^+^/CD11b^+^/F4/80^+^/CD206^+^ cells) in the kidneys between WT and OSMRβ^−/−^ mice on day 3, but total, M1, and M2 macrophages in OSMRβ^−/−^ mice were reduced compared with those in WT mice on day 6 (Fig. 5).

### Expressions of markers of kidney injury and fibrosis in the kidneys of OSMRβ^−/−^ mice

To assess the roles of OSM signaling in GOx-induced kidney injury and fibrosis, we investigated the expressions of genes related to kidney injury (kidney injury molecule-1, KIM-1), and fibrosis (transforming growth factor-β, TGF-β; type 1 collagen α2 chain, Col1a2; α-smooth muscle actin, αSMA; and tissue inhibitor of metalloproteinase 2, Timp2). The expressions of KIM-1, TGF-β, and Col1a2 were up-regulated by GOx injection in the kidneys of WT mice on day 3, but were decreased on day 6 (Fig. 3h–j). In addition, αSMA and Timp2 were increased on day 3 and day 6 compared with those on day 0 in WT mice (Fig. 3k,l). In the kidneys of OSMRβ^−/−^ mice on day 3, the expressions of KIM-1, TGF-β, Col1a2, and αSMA were significantly suppressed compared with WT mice (Fig. 3h–k). Western blot analysis confirmed the significant suppression of protein expressions of TGF-β and Col1a2 in OSMRβ^−/−^ mice on day 3 (Fig. 4i,j). On day 6, the expression of KIM-1 in OSMRβ^−/−^ mice was lower than that in WT mice (Fig. 3h). There were no significant changes in the expression of Timp2 mRNA in the kidneys between WT and OSMRβ^−/−^ mice on day 3 and day 6 (Fig. 3l).

### Localization of OSM and OSMRβ protein in the kidneys after GOx injection

**Figure 6:**
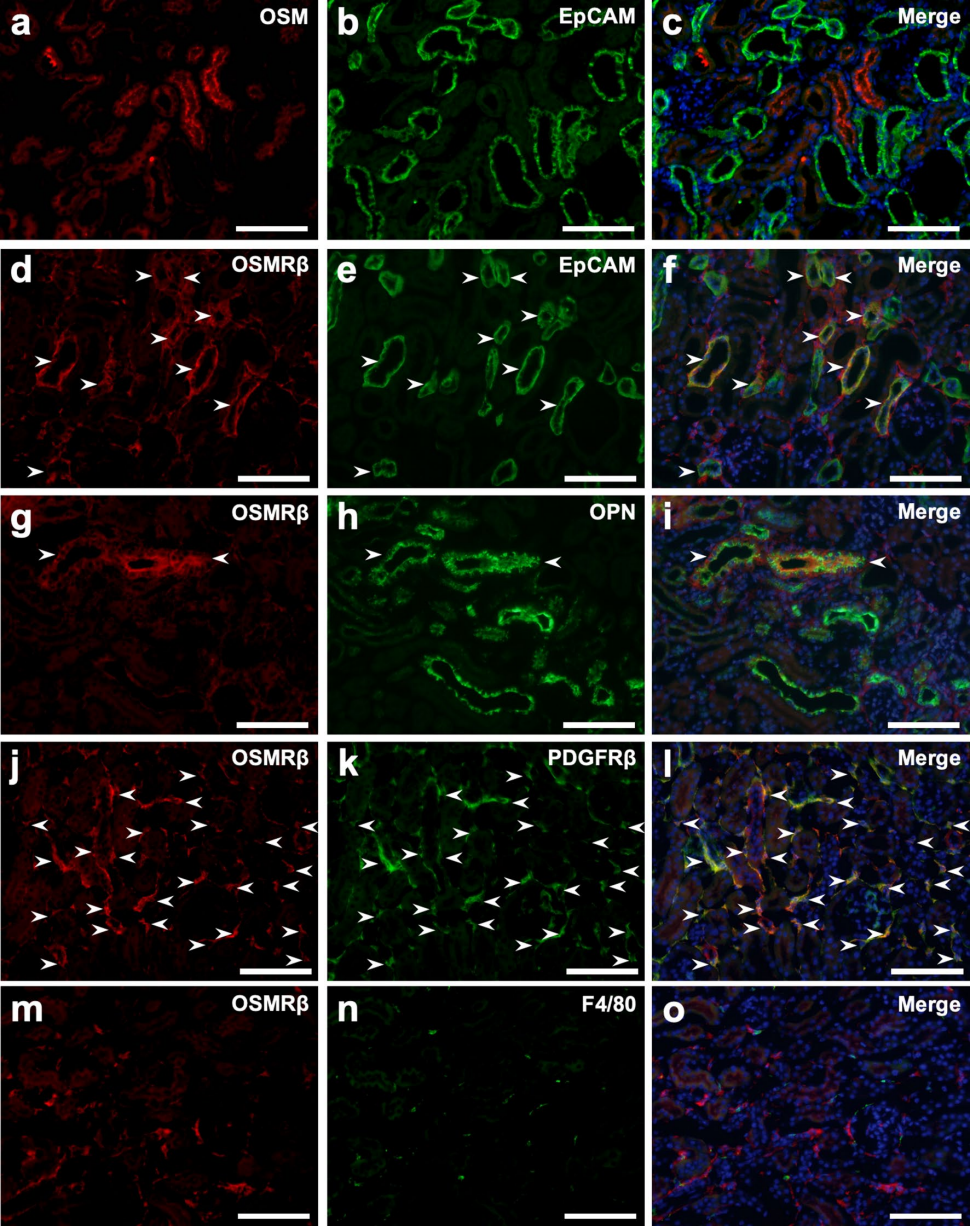
Localization of OSM and OSMRβ protein in the kidney. WT mice were given intraperitoneal injection of GOx (80 mg/kg) once daily for 3 days. Immunofluorescence staining for OSM (**a**) or OSMRβ (**d**, **g**, **j**, **m**) is shown in the left line (red). Immunofluorescence staining of EpCAM (**b**, **e**), OPN (**h**), PDGFRβ (**k**), and F4/80 (**n**) is shown in the middle line (green). The figures in the right line (**c**, **f**, **i**, **l**, **o**) show the merged images of panels in the left and middle lines. Nuclei were counterstained with DAPI (blue in **c**, **f**, **i**, **l**, **o**). Arrowheads indicate double-positive cells. Scale bars = 100 μm.

To identify OSM- and OSMRβ-expressing cells in the kidney of the mouse model of renal crystal formation, we performed immunofluorescence staining in the kidney of mice on day 3, when the renal expression of OSMRβ had reached a peak by the injection of GOx (Fig. 1). The expression of OSM was strongly observed in the EpCAM-negative renal tubular epithelial cells (RTECs) (Fig. 6a–c). In contrast, intense expression of OSMRβ was observed in the EpCAM-positive RTECs (Fig. 6d–f). These OSMRβ-positive RTECs expressed OPN (Fig. 6g–i). In addition, OSMRβ was expressed in platelet-derived growth factor receptor β (PDGFRβ)-positive fibroblasts (Fig. 6j–l). OSMRβ was not detected, however, in F4/80-positive macrophages (Fig. 6m–o). No expression of OSMRβ was detected in the kidneys of OSMRβ^−/−^ mice (Fig. S1).

### Direct effects of OSM on the RTECs and renal fibroblasts

**Figure 7:**
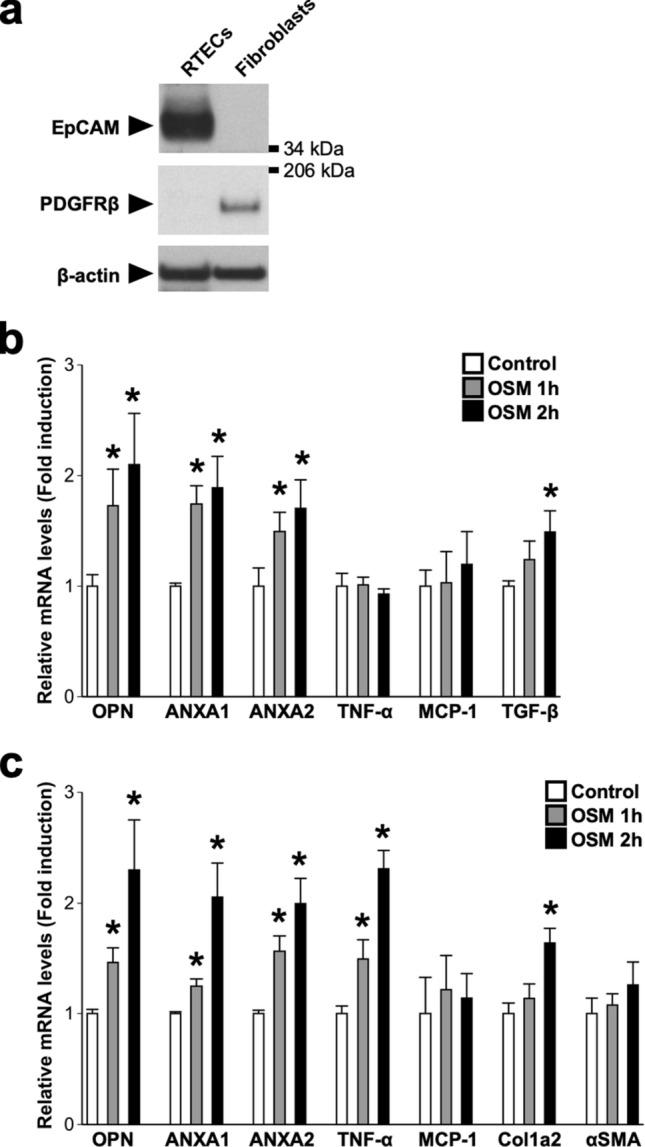
Direct effects of OSM on gene expressions in the RTECs and renal fibroblasts. RTECs and renal fibroblasts were isolated from the kidney of C57BL/6 J mice on day 3. Isolated cells were treated with OSM (50 ng/ml) for 1 or 2 h. (**a**) Western blot analysis of EpCAM and PDGFRβ in the RTECs and fibroblasts. The apparent molecular weights are indicated on the right. The full-length blots are presented in Supplementary Fig. S6. (**b**) Effects of OSM on the expressions of crystal-binding molecules (OPN, ANXA1, and ANXA2), inflammation-related genes (TNF-α and MCP-1), and fibrosis-related genes (TGF-β) in RTECs. (**c**) Effects of OSM on the expressions of crystal-binding molecules (OPN, ANXA1, and ANXA2), inflammation-related genes (TNF-α and MCP-1), and fibrosis-related genes (Col1a2 and αSMA) in renal fibroblasts. n = 4–6 per group. *P < 0.05 compared with controls.

**Figure 8:**
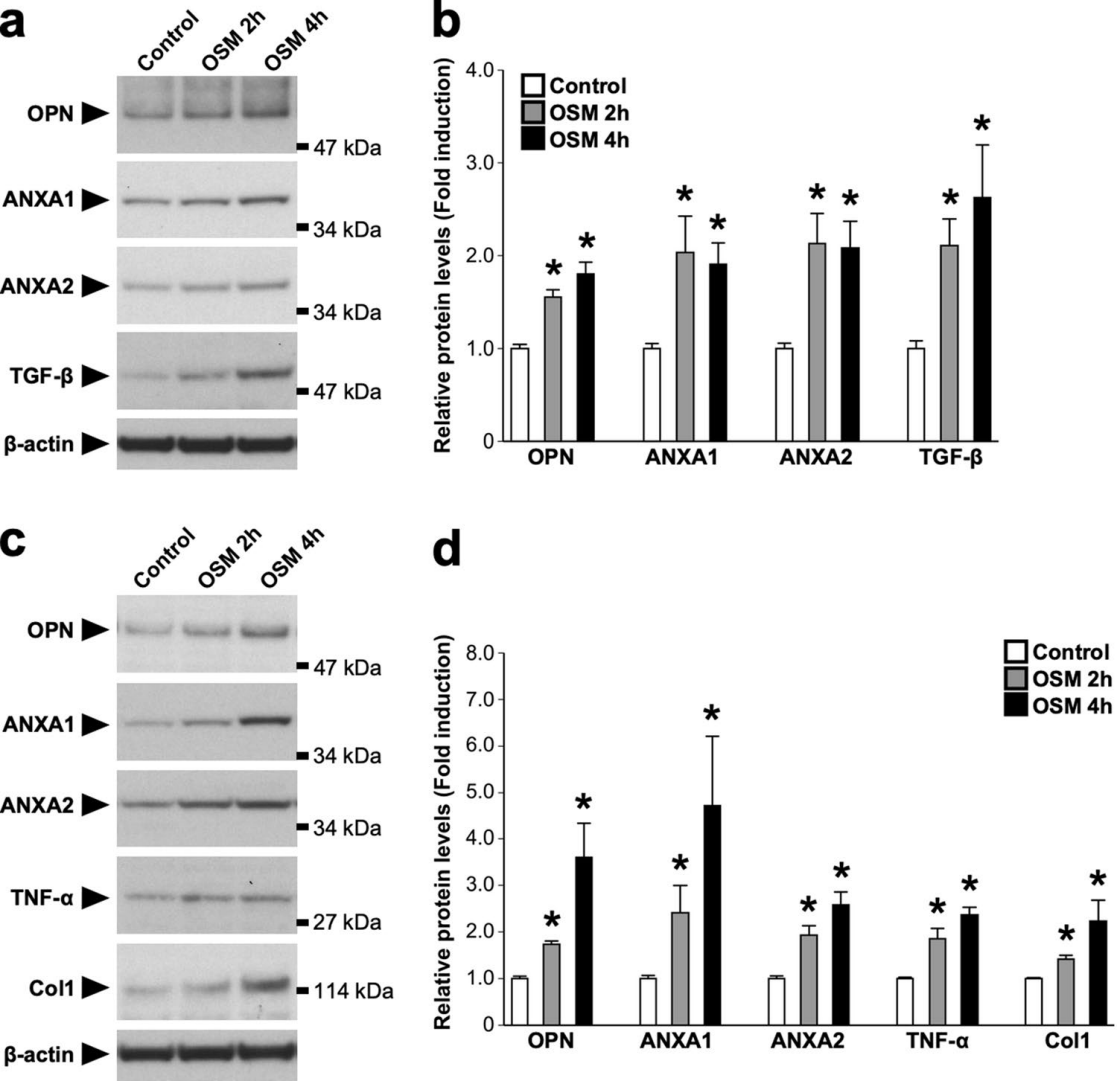
Direct effects of OSM on protein expressions in the RTECs and renal fibroblasts. RTECs and renal fibroblasts were isolated from the kidney of C57BL/6 J mice on day 3. Isolated cells were treated with OSM (50 ng/ml) for 2 or 4 h. (**a**) Western blot analysis of crystal-binding molecules (OPN, ANXA1, and ANXA2) and fibrosis-related molecules (TGF-β) in the OSM-stimulated RTECs. The apparent molecular weights are indicated on the right. (**b**) Quantitative analysis of the protein expressions of OPN, ANXA1, ANXA2, and TGF-β in the RTECs. The band intensities of OPN, ANXA1, ANXA2, and TGF-β were normalized to β-actin and are represented as the fold induction relative to the intensities of the controls (white bar) in the bar graph. (**c**) Western blot analysis of crystal-binding molecules (OPN, ANXA1, and ANXA2), inflammation-related molecule (TNF-α), and fibrosis-related molecules (Col1) in the OSM-stimulated renal fibroblasts. The apparent molecular weights are indicated on the right. (**d**) Quantitative analysis of the protein expression of OPN, ANXA1, ANXA2, TNF-α, and Col1 in the renal fibroblasts. The band intensities of OPN, ANXA1, ANXA2, TNF-α, and Col1 were normalized to β-actin and are represented as the fold induction relative to the intensities of the controls (white bar) in the bar graph. The full-length blots are presented in Supplementary Fig. S7. Data are expressed as mean ± SEM; n = 4 per group. *P < 0.05 compared with controls.

Finally, we checked the direct effects of OSM on the expressions of genes related to crystal adhesion, inflammation, and fibrosis in primary culture of RTECs and renal fibroblasts. The expressions of EpCAM and PDGFRβ were exclusively detected in the cell fractions we used as RTECs and renal fibroblasts, respectively (Fig. 7a). The mRNA expressions of OPN, ANXA1, and ANXA2 were increased by stimulation with OSM in both RTECs (Fig. 7b) and renal fibroblasts (Fig. 7c). In addition, OSM stimulation induced the expression of TNF-α mRNA in the renal fibroblasts (Fig. 7c), but not in RTECs (Fig. 7b). Stimulation with OSM did not affect the mRNA expression of MCP-1 in RTECs and renal fibroblasts (Fig. 7b,c). Furthermore, mRNA expressions of TGF-β (Fig. 7b) and Col1a2 (Fig. 7c) were induced by OSM in the RTECs and renal fibroblasts, respectively. The expression of αSMA, a marker of myofibroblasts, was not changed by the stimulation with OSM in renal fibroblasts (Fig. 7c). The effects of OSM on the expression of these molecules were abolished in RTECs and fibroblasts obtained from the kidneys of OSMRβ^−/−^ mice (Fig. S2). In addition, Western blot analysis confirmed the significant increases in protein expressions of these molecules in RTECs and renal fibroblasts stimulated with OSM (Fig. 8).

## Discussion

A member of the IL-6 family of cytokines, OSM, is associated with the development of a variety of human diseases, including rheumatoid arthritis^25^, metabolic syndrome^26^, and inflammatory bowel disease^22^. Although the expression of OSM increases in the kidneys of patients with obstructive nephropathy due to urolithiasis^27^, the functional roles of OSM in the development of kidney stone disease have been unclear. In the present study, we used a mouse model of renal crystal formation in OSMRβ^−/−^ mice to address this question. In the kidneys of OSMRβ^−/−^ mice, there was considerably less formation of GOx-induced crystal deposits than in WT mice on day 6, suggesting that OSM signaling may promote the formation of renal crystal deposits in the process of kidney stone formation.

Renal macrophages were recently reported to play an important role in the formation of crystal deposits. Generally, activated macrophages are divided into two types: classically activated macrophages (M1 macrophages) and alternatively activated macrophages (M2 macrophages)^28,29^. In the process of kidney stone formation, M1 macrophages promote crystal deposit formation by increases in the expressions of inflammatory cytokines and crystal-binding molecules^18^. We previously reported that OSM switches the phenotype of macrophage from M1 to M2 type in adipose tissue macrophages, peritoneal exudate macrophages, and a macrophage cell line, RAW264.7 cells^23^. Our initial hypothesis was therefore that OSM might inhibit kidney stone formation through decreases in M1 type of renal macrophages. In the present study, however, there were no significant differences in the number of M1 macrophages and in the expression of MCP-1 between WT and OSMRβ^−/−^ mice on day 3. MCP-1 is a chemokine that recruits monocytes to the site of injury. Moreover, the expression of OSMRβ was hardly detected in the renal macrophages. From these findings, OSM is suggested to have no association with the recruitment and phenotypic changes of renal macrophages in the process of kidney stone formation.

Crystal-binding molecules, such as OPN, ANXA1, and ANXA2, have been reported to be important for the crystal aggregation, crystal adhesion to RTECs, and crystal retention in renal tubules^30–32^. Experiments using cell lines of RTEC have shown that high calcium state and calcium oxalate crystals directly induce the expressions of these crystal-binding molecules^30,33,34^. Meanwhile, the mechanism of the production of crystal-binding molecules in the process of kidney stone formation is not fully understood. In the present study, OSM induced the expressions of OPN, ANXA1, and ANXA2, in RTECs isolated from the mouse model of renal crystal formation. In addition, our in vivo study showed that strong expression of OSMRβ was observed in the RTECs during renal crystal formation. The expressions of OPN, ANXA1, and ANXA2 induced by GOx injection were significantly lower in the kidneys of OSMRβ^−/−^ mice than in those of WT mice. OSM may therefore promote the formation of renal crystal deposits through the expression of crystal-binding molecules in the process of kidney stone formation.

In addition to the RTECs, OSMRβ was expressed in the renal fibroblasts. Fibroblasts are mainly responsible for the synthesis of extracellular matrix (ECM) proteins, such as collagen and elastin, in both physiological and pathological conditions^35^. In addition to these functions, fibroblasts have been reported to be associated with a variety of biological processes, including inflammatory and immune responses, angiogenesis, and carcinogenesis of adjacent epithelial cells^36–38^. Previously, Umekawa et al. reported that the expressions of OPN and MCP-1 are induced in NRK-49F cells, a normal rat kidney fibroblast-derived cell line, stimulated with calcium oxalate crystals^39^. In the present study, OSM induced the expressions of crystal-binding molecules, including OPN, ANXA1, and ANXA2, in the cultured renal fibroblasts. This suggests that OSM is a novel potent inducer of crystal-binding molecules in the fibroblasts as well as the RTECs. TNF-α was also induced by stimulation with OSM in the renal fibroblasts, while the expression of MCP-1 was not affected by OSM. From these findings, OSM seems to regulate inflammation through the production of inflammatory cytokines rather than by recruitment of M1 macrophages in the process of kidney stone formation.

Kidney stone disease is a risk factor for renal fibrosis and subsequent CKD^1–3^. In the process of renal fibrosis, TGF-β is important for the differentiation of renal fibroblasts into myofibroblasts, which are a main source of extracellular matrix (ECM) proteins, including collagens and fibronectin^40,41^. In the present study, on day 3, the expressions of TGF-β and αSMA, a marker of myofibroblasts, were lower in the kidneys of OSMRβ^−/−^ mice than those in WT mice. Suppression of Col1a2 expression was also observed in the kidneys of OSMRβ^−/−^ mice. In addition, in vitro study revealed that OSM directly induced the expression of TGF-β in the RTECs, while αSMA was not affected by OSM in the renal fibroblasts. These findings suggest that OSM produces TGF-β in RTECs and that the differentiation into myofibroblasts is indirectly induced. The expression of Col1a2, one of the ECM proteins related to fibrosis, was enhanced by OSM in the renal fibroblasts. OSM signaling thus promotes renal fibrosis in the process of kidney stone formation.

In humans, CaOx kidney stones mainly form as overgrowths on sub-epithelial plaques of calcium phosphate in the renal papillae, known as Randall’s plaque^1,3^. Several lines of evidence have suggested that CaOx crystals in the tubules and interstitium cause the formation of Randall’s plaque^1^. Several mouse models have been developed to study the mechanisms underlying the formation of CaOx crystals and subsequent Randall’s plaque. To date, there are no mouse models for Randall’s plaque, except for a mutant mouse lacking ABCC6 (ATP-binding cassette sub-family C member 6)^42^. However, mutations in ABCC6 are rarely responsible for kidney stone disease in humans^43^. Generally, single-gene mutations only account for 10–20% of kidney stone disease^44,45^, and the common pathogenesis of kidney stone disease is believed to involve the environmental and lifestyle factors. A mouse model of CaOx kidney stone disease using non-genetically engineered (normal) mice has therefore been considered to be suitable for investigation of the pathogenesis of human CaOx kidney stones. Okada et al.^46^ have established a mouse model forming CaOx crystals in normal mice (C57BL/6 mice) by intraabdominal injection of GOx^46^, and we used this model in the present study. Unfortunately, it is likely that the crystals will not develop into Randall’s plaque in this mouse model, as they disappeared by day 15^46^. However, intratubular crystals are grown within a very short time period, 3–6 days^46^, compared with the other mouse model using C57BL/6 mice, the high-oxalate/calcium-free diet-fed model, which needs 14 days to grow crystals^47^. The mouse model used in the present study is easy to prepare and may therefore be a good model for investigating the common pathogenesis of CaOx crystal formation in humans.

In the present study, we demonstrated that the deficiency of OSM signaling suppressed the formation of renal crystal deposits and OSM directly induced the production of crystal-binding molecules in the RTECs and renal fibroblasts. Our results strongly suggest that OSM contributes to the formation of renal crystal deposits through the induction of crystal-binding molecules in the process of kidney stone formation.

## Methods

### Animals

Eight-week-old male C57BL/6 J mice were purchased from Nihon SLC (Hamamatsu, Japan). Generation of OSMRβ^−/−^ mice followed the protocol as previously described^48^. OSMRβ^+/+^ (WT) and OSMRβ^−/−^ littermates were obtained from our breeding colony using heterozygous breeding pairs. All mice were housed in specific pathogen-free facilities under light [12-h (h) light/dark cycle]-, temperature (22–25 °C)-, and humidity (50–60% relative humidity)-controlled conditions. The mice had free access to food (MF; Oriental Yeast, Tokyo, Japan) and water. All experimental procedures were approved by the Animal Research Committees of Wakayama Medical University, and were carried out in accordance with the National Institutes of Health Guide for the Care and Use of Laboratory Animals (NIH publication No. 80-23, revised 1978) and the in-house guidelines for the care and use of laboratory animals of Wakayama Medical University.

### Injection of GOx in mice

As described elsewhere^46^, we prepared a mouse model of renal crystal formation by intraperitoneal injection of GOx (Sigma, St. Louis, MO). Briefly, mice were intraperitoneally injected with GOx dissolved in PBS once daily at a dose of 80 mg/kg. Day 0 was the first day of GOx injection and the “day 0” samples were taken from mice before the injection. Mice were injected with GOx once daily for 3, 6, or 9 days, and then sacrificed 24 h (h) after the final injection of GOx (the samples on day 3, 6, or 9, respectively). For example, the samples of “day 3” were taken from mice injected with GOx on day 0, day 1, and day 2, and sacrificed on day 3.

### Pizzolato staining and quantification of the amount of crystal deposits

The mice were deeply anaesthetized with isoflurane and transcardially perfused with ice-cold 0.9% NaCl followed by 4% paraformaldehyde. The kidney was then quickly removed, post-fixed in the same fixative at 4 °C for 4 h, and cryoprotected in 30% sucrose in 0.1 M PBS for 16 h. The specimens were embedded in an optimal cutting temperature (OCT) medium (Sakura Finetek, Torrance, CA), frozen rapidly in cold n-hexane on dry ice, and stored at -80 °C. Frozen sections were cut on a cryostat at 6-μm thickness. Pizzolato staining was performed to detect oxalate-containing crystals as described elsewhere^49^. Briefly, the solution for Pizzolato staining was prepared by a mixture of equal volumes of 5% silver nitrate and 30% hydrogen peroxidase. Frozen sections were incubated with the solution for 30 min (min). During the incubation, the sections were exposed to light from a 60-W incandescent lamp at a distance of 15 cm. The sections were then washed with distilled water and counterstained with nuclear fast red (Vector Laboratories, Burlingame, CA). Whole images of the kidney sections were acquired on a light microscope (BZ-7000, Keyence, Tokyo, Japan) using the image stitching system. The total area and positive area by Pizzolato staining in each kidney section were measured using Image J (US National Institutes of Health, Bethesda, MD). Positive area by Pizzolato staining was normalized by the total area of the kidney section. Coronal sections of the kidney containing the cortex, medulla, and papilla, were used for quantification of crystal deposits. Six sections were selected at 120 μm intervals from the midcoronal plane of each kidney.

### Immunofluorescence staining

Immunofluorescence staining was performed as described previously^23^. Briefly, the mice were deeply anaesthetized with isoflurane and transcardially perfused with ice-cold 0.9% NaCl followed by ice-cold modified Zamboni’s fixative (2% paraformaldehyde and 0.2% picric acid in 0.1 M PBS). The kidney was quickly removed and postfixed in the same fixative at 4 °C for 3 h. All specimens were then immersed in 20% sucrose in 0.1 M PBS for 16 h. The specimens were embedded in an OCT medium, frozen rapidly in cold n-hexane in dry ice, and stored at -80 °C. Frozen sections were cut on a cryostat at 6-μm thickness. The sections were preincubated with 5% normal donkey serum at room temperature for 1 h, followed by incubation with primary antibodies at 4 °C for 16 h. The primary antibodies were used at the following dilution: goat anti-OSM antibody (diluted at 1:100; ab10843, Abcam, Cambridge, UK), goat anti-OSMRβ antibody (1:200; AF662, R&D Systems, Minneapolis, MN), rabbit anti-EpCAM antibody (1:100; ab71916, Abcam), rabbit anti-OPN antibody (1:100; ab218237, Abcam), rabbit anti-PDGFRβ antibody (1:100; ab91066, Abcam), and rat anti-F4/80 antibody (1:50; clone A3-1, Serotec, Oxford, UK). The sections were then incubated with Cy2-conjugated or Cy3-conjugated secondary antibodies (Jackson ImmunoResearch) at room temperature for 1 h. The sections were counterstained with 4′, 6-diamino-2-phenylindole (DAPI). Immunofluorescence images were acquired using an epifluorescence microscope (Olympus, Tokyo, Japan) equipped with a digital charge-coupled device camera (Olympus).

### Preparation of single cell suspensions from mouse kidney

The mice were deeply anesthetized with isoflurane and the kidney was quickly removed. The kidney was minced into fine pieces and digested with collagenase type II (Sigma) dissolved in Dulbecco's modified Eagle's medium (DMEM; Invitrogen, Carlsbad, CA) supplemented with 2% fetal calf serum (FCS) using a gentleMACS Dissociator (Miltenyi Biotec, Bergisch Gladbach, Germany). The samples were then passed through a nylon mesh (100-μm pore size; BD Biosciences, San Jose, CA) and centrifuged at 1200 rpm for 5 min. The cells in the pellets were resuspended in DMEM supplemented with 2% FCS and used for the analysis of flow cytometry and the isolation of RTECs and renal fibroblasts.

### Flow cytometry

Flow cytometry was performed as described previously^23^. Briefly, the cells isolated from the kidney were incubated with anti-CD16/CD32 antibodies (1:100, BD Biosciences) to block Fc binding at 4 °C for 5 min, followed by incubation with fluorescently-labeled primary antibodies or isotype-matched control antibodies at 4 °C for 30 min. The FITC-conjugated anti-CD45 antibody (clone 30-F11), phycoerythrin (PE)-conjugated anti-CD11b antibody (clone M1/70), APC-conjugated anti-F4/80 antibody (clone BM8), PE-Cy7-conjugated anti-Ly6C antibody (clone HK1.4), and PE-Cy7-conjugated anti-CD206 antibody (clone MR6F3) were purchased from eBiosciences (San Diego, CA). The stained cells were analyzed using a BD FACSVerse flow cytometer (BD Biosciences). Dead cells were removed from the analysis using 7-aminoactinomycin-D (7-AAD) staining (eBioscience). The results of flow cytometry were analyzed with FlowJo software (Tree Star, Ashland, OR). The plot of a forward- versus side-scatter was used as the first gate to gate out aggregates and debris (Fig. S3). To identify individual live cells, the events were then gated based on side scatter versus 7-AAD (Fig. S3). Next, the CD45, F4/80, and CD11b-triple-positive cells were selected as macrophages in the kidney (Fig. S3). M1 and M2 macrophages were identified as Ly6C- and CD206-positive cells, respectively, in the macrophage fraction as previously described^17^. Single color controls were used to set the compensation and gates.

### Isolation of RTECs and renal fibroblasts from kidney

First, the cells obtained from kidney were incubated with anti-CD16/CD32 antibodies to block Fc binding. For the isolation of RTECs, the cells were incubated with APC-conjugated anti-EpCAM antibody (clone G8.8, eBioscience). The stained cells were sorted using the anti-APC Multisort kit (Miltenyi Biotec) and the autoMACS Pro Separator (Miltenyi Biotec). The sorted cells were cultured in DMEM supplemented with 10% FCS, 100 U/ml of penicillin (Invitrogen), and 100 μg/ml of streptomycin (Invitrogen) for three days, and used as RTECs.

According to the previously reported method for the isolation of renal fibroblasts^50^, the cells isolated from the kidney were plated on the collagen-coated six-well plates (Corning, Corning, NY) in DMEM supplemented with 10% FCS, 100 U/ml of penicillin (Invitrogen), and 100 μg/ml of streptomycin (Invitrogen). The cells were then passaged at confluence and the fourth passage cells were used as renal fibroblasts.

These cells were then treated with vehicle or 50 ng/ml of recombinant mouse OSM (R&D Systems) and maintained for 1 and 2 h. All cells were cultured at 37 °C in a humidified atmosphere of 5% CO_2_.

### Western blot analysis

A Western blot analysis was performed with some modifications, as previously described^23^. Lysates from the kidneys of the mice and the cultured cells were prepared using a RIPA buffer (Upstate Biotechnology, Lake Placid, NY) containing a protease inhibitor cocktail (Upstate Biotechnology), 1 mM orthovanadate, 1 mM sodium fluoride, and 1 mM phenylmethylsulfonyl fluoride. The protein concentrations of the lysates were determined using the BCA Protein Assay kit (PIERCE, Rockford, IL). Sodium dodecyl sulfate–polyacrylamide gel electrophoresis was used to separate 20 μg of protein from the kidneys and 10 μg of protein from the cultured cells before transfer to PVDF membranes (GE Healthcare, Little Chalfont, UK). The blotted membranes were incubated with goat anti-OSM antibody (diluted at 1:100, Abcam), goat anti-OSMRβ antibody (diluted at 1:500, R&D Systems), goat anti-OPN antibody (diluted at 1:500, Santa Cruz Biotechnology, Santa Cruz, CA), rabbit anti-ANXA1 antibody (diluted at 1:1000, Abcam), rabbit anti-ANXA2 antibody (diluted at 1:1000, Abcam), rabbit anti-TNF-α antibody (diluted at 1:500, Abcam), rabbit anti-IL-1β antibody (diluted at 1:500, Santa Cruz Biotechnology), goat anti-MCP-1 antibody (diluted at 1:500, R&D Systems), rabbit anti-TGF-β1 antibody (diluted at 1:500, Abcam), biotinylated rabbit anti-collagen I antibody (diluted at 1:500, Rockland), rabbit anti-EpCAM antibody (diluted at 1:1000, Abcam), and rabbit anti-PDGFRβ antibody (diluted at 1:500, Abcam). Thereafter, the membranes were incubated with HRP-conjugated donkey anti-rabbit antibody (diluted at 1:4000, GE Healthcare), HRP-conjugated donkey anti-goat antibody (diluted at 1:10,000, Jackson ImmunoResearch), or HRP-conjugated streptavidin (diluted at 1:200, SeraCare Life Sciences, Milford, MA). Labeled proteins were detected with chemiluminescence using the ECL detection reagent (GE Healthcare) according to the manufacturer's instructions. The membranes were exposed to hyperfilm ECL (GE Healthcare) for an appropriate period. Subsequently, the blotted membranes were stripped in 0.25 M glycine, pH 2.5, at RT for 10 min and incubated with mouse anti-β-actin antibody (diluted at 1:10,000; Sigma), followed by incubation with HRP-conjugated donkey anti-mouse IgG antibody (diluted at 1:20,000, GE Healthcare).

### Quantitative real-time PCR

Quantitative real-time PCR was performed with some modifications as described previously^23^. Briefly, total RNAs from the kidney, cultured RTECs, and cultured renal fibroblasts were prepared using TRI reagent (Molecular Research Center, Cincinnati, OH). Using the total RNA, the cDNA was synthesized with High Capacity cDNA Reverse Transcription Kit (Applied Biosystems, Foster City, CA). The following TaqMan Gene Expression Assays (Applied Biosystems) were used: OSM (Mm01193966_m1), OSMRβ (Mm00495424_m1), F4/80 (Mm00802529_m1), TNF-α (Mm00443258_m1), IL-1β (Mm00434228_m1), MCP-1 (Mm00441242_m1), OPN (Mm00436767_m1), ANXA1 (Mm00440225_m1), ANXA2 (Mm01150673_m1), KIM-1 (Mm00506686_m1), TGF-β (Mm01178820_m1), Col1a2 (Mm00483888_m1), αSMA (Mm00725412_m1), Timp2 (Mm00441825_m1), and 18S (Hs99999901_s1). Quantitative real-time PCR for each gene was performed using Rotor Gene Q (QIAGEN, Hilden, Germany) and Rotor Gene Probe PCR Kits (QIAGEN). The PCR amplification protocol was 95 °C for 10 min and then 40 cycles of 95 °C for 10 s (sec) and 60 °C for 45 s. The expression of each gene was normalized by 18S ribosomal RNA expression and analyzed using ΔΔCT method.

### Statistical analysis

Results are shown as mean ± SEM. Comparison between the two groups was analyzed by Student’s *t*-test. For multiple group comparisons, ANOVA followed by the post hoc Bonferroni test was used. For all statistical tests, the significance threshold was *p* < 0.05.

## Supplementary information

Supplementary file1

## Data Availability

All authors had full access to all the data in the study and take responsibility for the integrity of the data and the accuracy of the data analysis.
