# Supplementary material for: Essential roles of oncostatin M receptor β signaling in renal crystal formation in mice
Source: Sci Rep. 2020 Oct 13;10:17150. doi: 10.1038/s41598-020-74198-3 (PMC7553912; doi:10.1038/s41598-020-74198-3)
Supplement: Supplementary file 1 — Supplementary file1 [file 41598_2020_74198_MOESM1_ESM.docx]

**Supplementary Information**

Essential roles of oncostatin M receptor β signaling in renal crystal formation in mice

Shimpei Yamashita^1^, Tadasuke Komori^2^, Yasuo Kohjimoto^1^, Atsushi Miyajima^3^, Isao Hara^1^, Yoshihiro Morikawa^2^

Department of Urology, Wakayama Medical University, Wakayama, Japan^1^

Department of Anatomy & Neurobiology, Wakayama Medical University, Wakayama, Japan^2^

Laboratory of Cell Growth and Differentiation, Institute for Quantitative Biosciences, The University of Tokyo, Tokyo, Japan^3^

Corresponding author: Yoshihiro Morikawa, Department of Anatomy & Neurobiology, Wakayama Medical University, 811-1 Kimiidera, Wakayama 641-8509, Japan. Tel. & Fax: +81-73-441-0617. E-mail address; [yoshim@wakayama-med.ac.jp](mailto:yoshim@wakayama-med.ac.jp)


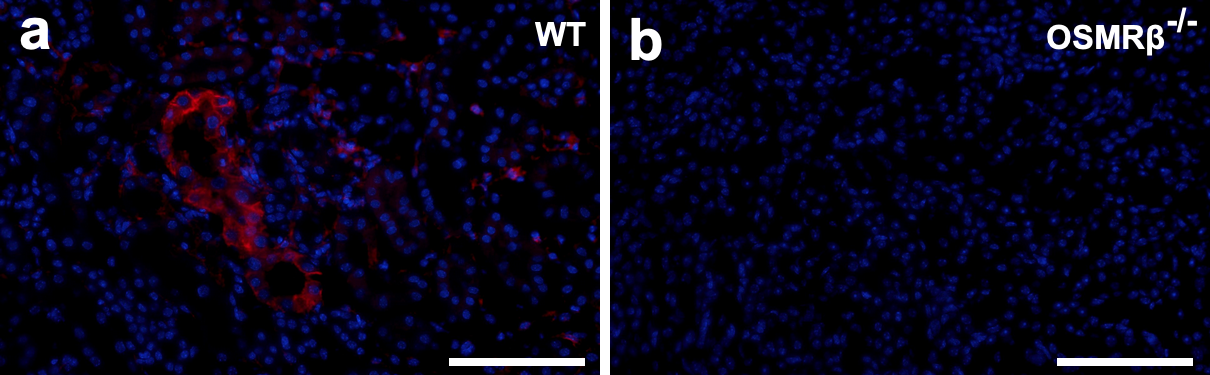
Supplementary Figure S1. The specificity of anti-OSMRβ antibody. Immunohistochemistry of OSMRβ by using goat anti-OSMRβ antibody in the kidneys of WT and OSMRβ^-/-^ mice (n = 3 per group). Immunoreactivity for OSMRβ was observed in the kidney of WT (a), but not in the kidney of OSMRβ^-/-^ mice (b). Nuclei were counterstained with DAPI. Scale bars = 100 μm



Supplementary Figure S2. Effects of OSM on gene expressions in the RTECs and renal fibroblasts obtained from the kidney of OSMRβ^-/-^ mice. RTECs and renal fibroblasts were isolated from the kidney of GOx (80 mg/kg)-injected OSMRβ^-/-^ mice on day 3. Isolated cells were treated with OSM (50 ng/ml) for 1 or 2 hrs. (a) Effects of OSM on the expressions of crystal-binding molecules (OPN, ANXA1, and ANXA2) and fibrosis-related genes (TGF-β) in RTECs (EpCAM^+^ cells). (b) Effects of OSM on the expressions of crystal-binding molecules (OPN, ANXA1, and ANXA2), inflammation-related genes (TNF-α), and fibrosis-related genes (Col1a2) in the renal fibroblasts. n = 4 per group.


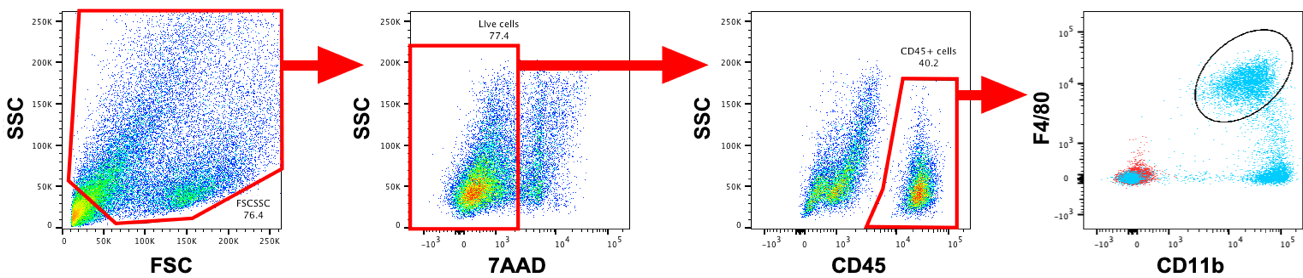


Supplementary Figure S3. Identification of total macrophages in the kidney by flow cytometry. Cells were isolated with collagenase treatment of the kidney and labeled with antibodies against CD45 (FITC), CD11b (PE), and F4/80 (APC). Positive staining was identified using isotype control- and single-stained samples. First, cell debris was excluded from the analysis using a polygon gate (red) in the FSC/SSC plot (left panel). Second, a rectangle gate (red) in the 7AAD/SSC plot was used to eliminate dead cells (second panel from the left). Third, the CD45-positive cells were gated using a polygon gate (red) in the CD45/SSC plot to select the leukocyte population (third panel from the left). Total macrophages were gated using an ellipsoid gate (black) in the CD11b/F4/80 plot in the leukocyte population (right panel).


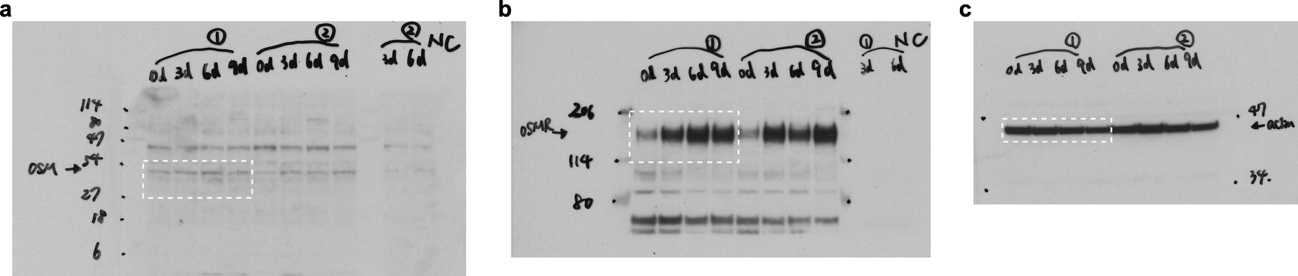
Supplementary Figure S4. Full-length blots of the data shown in Figure 1c. Full-length blots of OSM (a), OSMRβ (b), and β-actin (c) are shown. The cropped regions shown in Figure 1c are outlined by white dashed line in each panel.


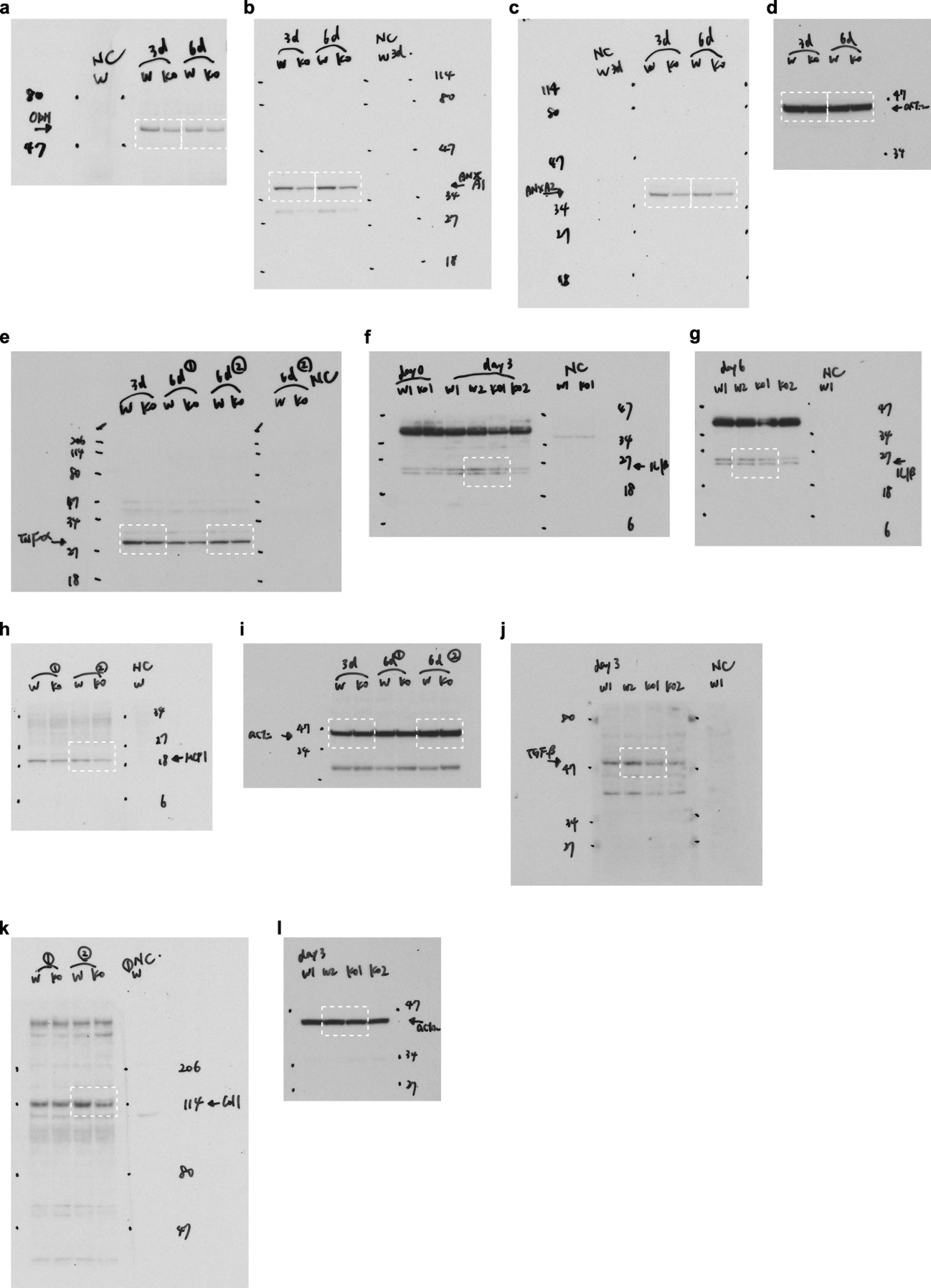


Supplementary Figure S5. Full-length blots of the data shown in Figure 4. Full-length blots of OPN (a; day 3 and day 6), ANXA1 (b; day 3 and day 6), ANXA2 (c; day 3 and day 6), TNF-α (e; day 3 and day 6), IL-1β (f; day 3, g; day 6), MCP-1 (h; day 6), TGF-β (j; day 3), Col1 (k; day 3), and β-actin (d, i, l) are shown. The cropped regions shown in Figure 4 are outlined by white dashed line in each panel.


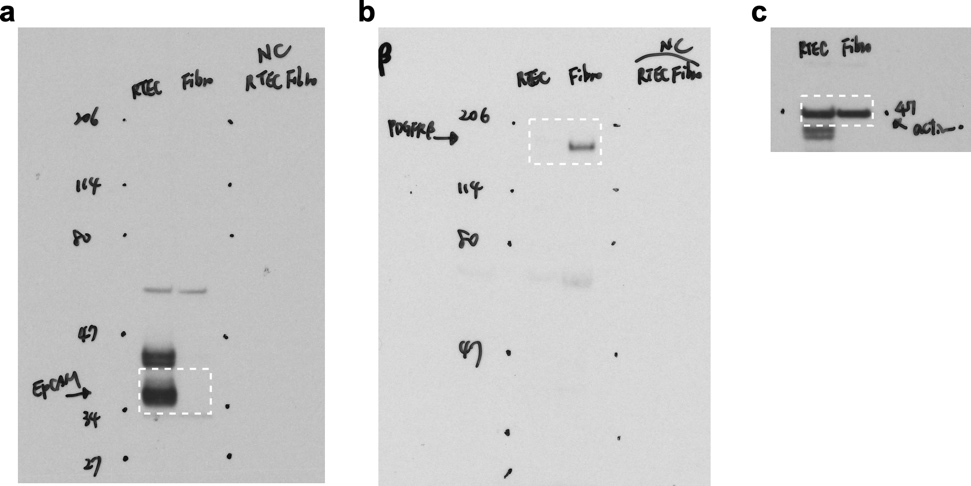
Supplementary Figure S6. Full-length blots of the data shown in Figure 7a. Full-length blots of EpCAM (a), PDGFRβ (b), and β-actin (c) are shown. The cropped regions shown in Figure 7a are outlined by white dashed line in each panel.


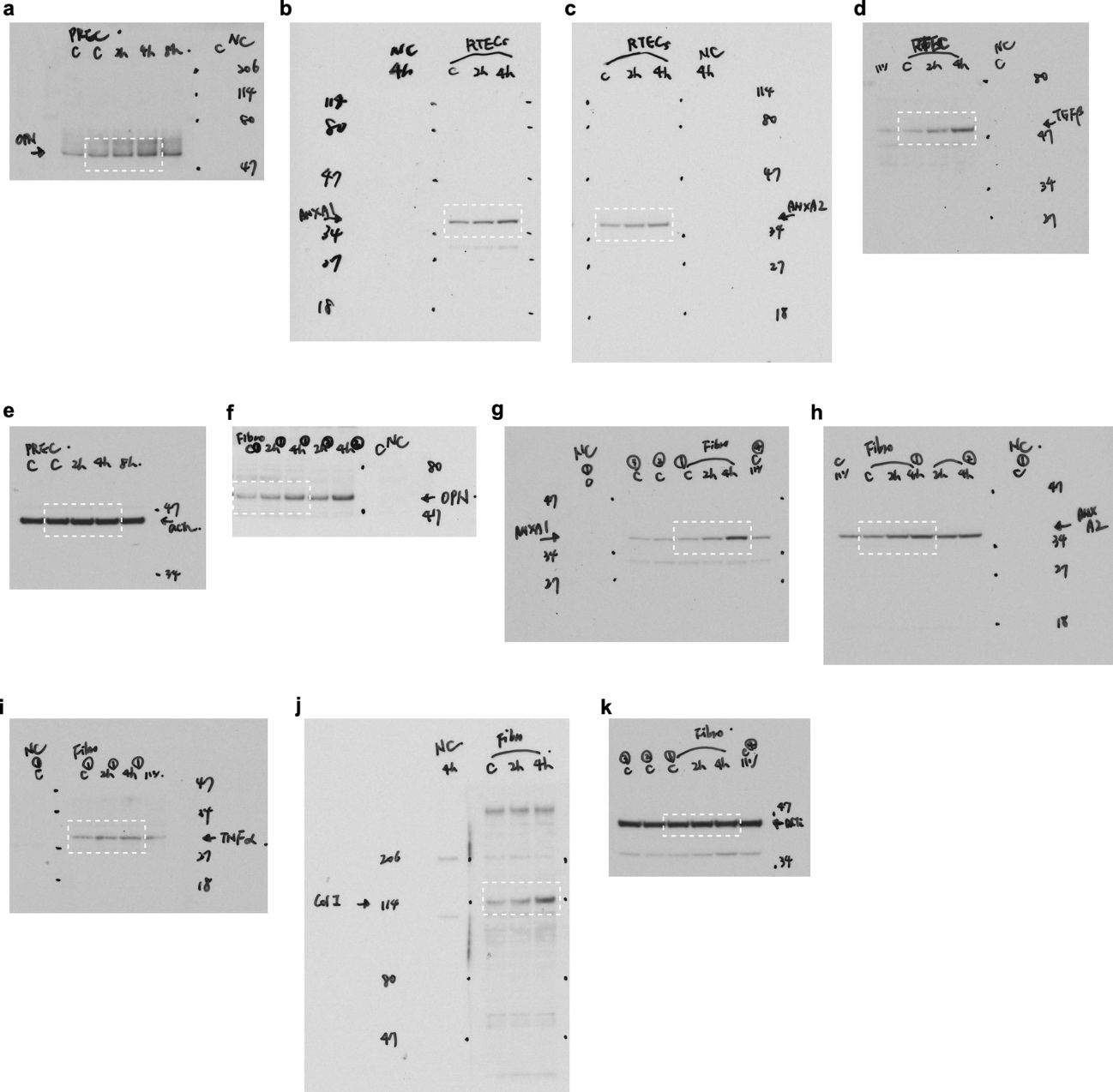


Supplementary Figure S7. Full-length blots of the data shown in Figure 8. Full-length blots of OPN (a; RTECs, f; renal fibroblasts), ANXA1 (b; RTECs, g; renal fibroblasts), ANXA2 (c; RTECs, h; renal fibroblasts), TGF-β (d; RTECs), TNF-α (i; renal fibroblasts), Col1 (j; renal fibroblasts), and β-actin (e; RTECs, k; renal fibroblasts) are shown. The cropped regions shown in Figure 8 are outlined by white dashed line in each panel.
